# Physicochemical Characterization and Antioxidant Properties of Essential Oils of *M. pulegium* (L.), *M. suaveolens* (Ehrh.) and *M. spicata* (L.) from Moroccan Middle-Atlas

**DOI:** 10.3390/foods12040760

**Published:** 2023-02-09

**Authors:** Nadia Zekri, Hanane Elazzouzi, Atika Ailli, Aman Allah Gouruch, Fatima Zahrae Radi, Mohammed Alaoui El Belghiti, Touriya Zair, Gema Nieto, Juan A. Centeno, José M. Lorenzo

**Affiliations:** 1 Laboratory of Spectroscopy, Molecular Modeling, Materials and Nanomaterials, Water and Environment, Department of Chemistry, Faculty of Sciences, University Mohammed V, 4- Avenue Ibn Battouta, Rabat 1014 PR, Morocco; 2 Research Team of Chemistry, Bioactive Molecules and Environment, Laboratoire des Matériaux Innovants et Biotechnologie des Ressources Naturelles, Department of Chemistry, Faculty of Sciences, University Moulay Ismail, Zitoune, Meknes 11201, Morocco; 3 Department of Food Technology, Food Science and Nutrition, Faculty of Veterinary Sciences, Regional Campus of International Excellence “Campus Mare Nostrum”, 30071 Espinardo, Spain; 4 Area de Tecnoloxía dos Alimentos, Facultade de Ciencias, Universidade de Vigo, 32004 Ourense, Spain; 5 Centro Tecnológico de la Carne de Galicia, Rúa Galicia n 4, Parque Tecnológico de Galicia, San Cibrao das Viñas, 32900 Ourense, Spain

**Keywords:** *Mentha*, essential oils, physicochemical properties, antioxidant activity, DPPH^•^ assay

## Abstract

The cosmetics and food fields are based on the use of synthetic substances to protect their products against oxidation. However, synthetic antioxidants were reported to have negative effects on human health. The interest to develop natural antioxidants from plants has been growing in recent decades. The aim of this study was to determine the antioxidant properties of three essential oils (EOs) of *M. pulegium* (L.), *M. suaveolens* (Ehrh.) and *M. spicata* (L.) from the Azrou and Ifrane regions. The organoleptic characteristics, yields and physical properties were determined for the selected EOs. Their chemical compositions were identified using GC-MS; then, their antioxidant activities were evaluated using the DPPH^•^ free radical scavenging activity and were compared with the ascorbic acid standard. The determined physicochemical parameters of dry matter and EOs demonstrated their good quality. The analysis of the EOs showed the dominance of pulegone (68.86–70.92%) and piperitenone (24.81%), piperitenone oxide (74.69–60.3%), and carvone (71.56–54.79%) and limonene (10.5–9.69%) for *M. pulegium*, *M. suaveolens* and *M. spicata*, respectively, from Azrou and Ifrane. Additionally, the antiradical tests demonstrated the remarkable power of these EOs, especially *M. pulegium* EO (IC_50_ = 15.93 mg/mL), which recorded the best activity compared with ascorbic acid (IC_50_ = 8.849 mg/mL). The obtained results indicated that these EOs could be applied as natural antioxidants in the food industry.

## 1. Introduction

Antioxidants are substances that the body produces as a reaction to environmental and other pressures in order to prevent or decelerate cell damage caused by free radicals [1]. Free radicals can also be naturally produced by an organism during biosynthetic processes, such as vitamins C (ascorbic acid) and E, carotenoids and phenolic compounds, or they can be synthetic [2]. Synthetic antioxidants, such as BHA and BHT, are widely used in the food and cosmetics industries because of their chemical stability and availability relative to natural ones [3]. However, they are now suspected to cause potential risks to human health, such as cardiovascular disease and cancer [4].

Consequently, great attention has been given to the natural antioxidants of plant extracts, especially to the essential oils that are known to possess potential as natural agents for food conservation. Recently, several EOs were qualified as natural antioxidants and proposed to replace synthetic ones in specific areas of food preservation [5,6]. The genus *Mentha* is characterized by the diversity of its secondary metabolites used as traditional medicines, food additives and household remedies for gastrointestinal disorders. They are also used as mouth fresheners, antibacterial agents, tonics, astringents, mild laxatives and culinary herbs [7].

*M. pulegium* (L.), *M. suaveolens* (Ehrh.) and *M. spicata* (L.) are species that are widely used by the Moroccan population in traditional medicine and for the richness of their EOs in active principles required in the food and pharmaceutical industries. In this context, we aimed in this research to study the physicochemical properties of dry matter and the EOs of selected mints due to the scarcity of previous works dealing with them to show the effect of the origin region on the yields and constituents of the EOs and to evaluate their capacity to scavenge the DPPH^•^ free radical.

## 2. Materials and Methods

### 2.1. Plant Material

**Figure 1:**
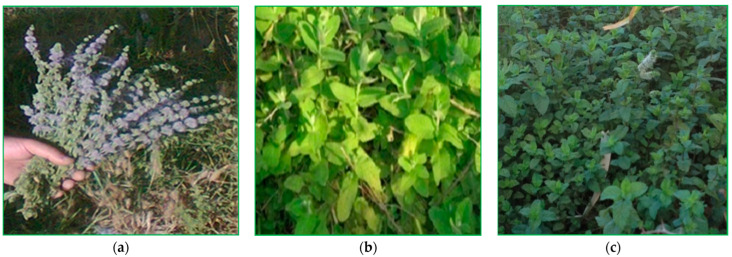
Morphological aspect of three selected *Mentha* species: (**a**) *M. pulegium* (L.), (**b**) *M. suaveolens* (Ehrh.) and (**c**) *M. spicata* (L.).

The aerial parts (leaves and flowers) of three *Mentha* species (Lamiaceae), namely, *M. pulegium* (L.), *M. suaveolens* (Ehrh.) and *M. spicata* (L.), were collected from two sites of the Middle Atlas: Azrou (latitude: 33°25′59″; longitude: 5°13′01″; altitude: 1278 m) and Ifrane (latitude: 31°42′07″; longitude: 6°20′57″; altitude: 2019 m) (Figure 1). The Middle Atlas is characterized by a semi-humid climate with strong continental influence and an annual average temperature of approximately 20 °C. All samples were spread in thin layers and then subjected to drying in the shade under conditions laboratory. The temperature varied from 12.85 to 38.25 °C during the drying period. The identification of the selected species was performed at the Department of Botany (Scientific Institute of Rabat).

### 2.2. Phytochemical Study

#### 2.2.1. Quality Control of the Dry Matter

##### Moisture Content

The moisture content (MS) was obtained by heating 5 g of the test sample, previously placed in dried crucibles, in an oven at 103 ± 2 °C [8]. The solid crucibles were weighed before and after drying. After 24 h, the MS was calculated and expressed as a percentage according to the following formula:

MC% = ((M_0_ − M_1_)/M_0_) × 100

M_0_: initial mass of the plant;
M_1_: mass after drying.

##### Determination of the pH

The procedure was performed according to NF V 05-108(1970) [9]. It defines the acidity of the product considered, measured with a pH meter. Ten milliliters of hot distilled water was added to 2 g of the sample. The mixture was crushed, filtered and allowed to cool. The electrode was immersed in a large volume of this filtrate and the pH value was noted. The electrode should be rinsed with distilled water before and after each measurement.

##### Ash Content

The ash content was determined according to NF V 05-113, 1972 [10]. The sample (4 g) was calcined at 550 °C in porcelain capsules. The capsules were placed in a muffle furnace until whitish ash of constant weight was obtained. They were removed from the oven and left to cool in the desiccator, then weighed. The organic matter was calculated first to deduce the ash percentage according to the following equations:

OM% = ((W_1_−W_2_)/Ts) × 100

OM%: organic matter;
W_1_: weight of the capsule and sample before calcination;
W_2_: weight of the capsule and the sample after calcination;
T_S_: test sample.

Then:

Ash % = 100 − OM%

##### Determination of the Acidity

According to NF V 05-101 [11], the acidity was obtained by adding 10 g of powder to 50 mL of boiling distilled water; the solution was then put in a water bath for 30 min. After cooling, the mixture was made up to 100 mL with distilled water and filtered; a few drops of phenolphthalein were added to 10 mL of the recovered filtrate. Thereafter, the titration was carried out with KOH until the persistent pink color changed. The acidity was determined using the following equation:

A% = ((250 V_1_ × 100)/(V_0_ M × 10)) × 0.07

M: mass in grams of the product taken;
V_0_: volume in milliliters of the test sample;
V_1_: volume in milliliters of the potassium hydroxide solution at the 0.1 N used.

#### 2.2.2. Quality Control of the Mint Species EOs

##### Determination of the Brix Degree

The Brix degree principle was based on measuring the concentration (%) of all solids (salts, sugar, proteins, fatty acids, etc.) dissolved in the oil using a refractometer device.

##### Determination of the Acid Value (AV)

For measuring the acidity of an EO, 1 g of the EO was dissolved in 5 mL of 96% alcohol, and a few drops of 1% phenolphthalein solution were then added. The mixture obtained was titrated using 0.1 N KOH solution (diluted in 96% ethanol) until a pale pink color appeared for 30 sec [12]. The following equation was then used to calculate the acid value:

AV = (VKOH 0.1 × 56.1)/Mass of essential oil

##### Determination of the Iodine Value

The iodine value was determined as described by Manuranjani et al. [13], where 0.5 g of the oil was taken for the analysis. The final solution was titrated with 0.1 N sodium thiosulphate using a starch indicator. The iodine value was then calculated using the following equation:

Iodine value = [(B–S) × N × 12.69]/weight of the sample

where:

B: volume (mL) of 0.1 N sodium thiosulphate required by blank;
S: volume (mL) of 0.1 N sodium thiosulphate required by sample;
N: normality of sodium thiosulphate solution.

##### Determination of the Peroxide Index

The peroxide index (PV) provides valuable information on the oxidation state of essential oils. For this test, about 1.0 g of the oil sample was taken and 1.0 g of KI and 20 mL of the solvent mixture (glacial acetic acid/chloroform, 2/1, *v*/*v*) were added to it. The mixture was then boiled for one minute. In a flask containing 20 mL of 5% KIO_3_ solution, the hot mixture solution was poured. A few drops of the starch solution were added to the mixture and titrated with 0.025 M sodium thiosulphate solution [13]. The following equation was then used to calculate the peroxide index:

PV (m Eq/kg) = (titre × N/100) × weight of the sample

N: normality of sodium thiosulphate solution.

#### 2.2.3. Extraction and GC-MS Analysis of the Mint EOs

The extraction of essential oils was conducted via hydrodistillation of the mint aerial parts (100 g) using a Clevenger apparatus over 3 h. The isolated EOs were dehydrated with anhydrous sodium sulfate and stored in a refrigerator at a temperature of 4 °C until use. For the calculation of the yields, three replicates were performed for each mint and the following formula was used:

Yield (%): (W(EO)/W_O_) × 100

W(EO): weight of the recovered EO (g);
W_O_: weight of the plant material (100 g).

The chromatographic analyses were performed using a Hewlett Packard gas chromatograph (HP 6890 series) equipped with an HP-5 capillary column (30 m × 0.25 mm × 0.25 microns’ film thickness) and an FID detector set at 250 °C; the setup was fed with a gas mixture of H_2_/air. The mode of injection was split; the carrier gas used was nitrogen with a flow rate of 1.7 mL/min. The column temperature was programmed at a mounted rate of 4 °C/min from 50 to 200 °C for 5 min. The unit was controlled by a computer system type “HP ChemStation” to manage the operation of the device and monitor the chromatographic analyses. GC-MS was carried out using a chromatograph Hewlett Packard (HP 6890) coupled to a mass spectrometer (HP 5973 series). Fragmentation was performed via electron impact at 70 eV. The used column was a capillary-type HP 5SM (30 m × 0.25 mm × 0.25 mm). The column temperature was programmed at a mounted rate of 4 °C/min from 50 to 200 °C for 5 min. The carrier gas was helium with a flow rate set at 1.7 mL/min. The injection mode was split type. 

For the compound identification, the Kovàts index of each compound was calculated in relation to the retention time of a series of linear alkanes (C_7_–C_40_). The calculated index was then compared with those of the Adams reference. The mass spectra of compounds were also matched with those stored in the NIST library/EPA/NIH MASS SPECTRAL LIBRARY; Version 2.0, 2002 (Thermo Scientific TSQ 8000 Evo, Waltham, MA, USA).

### 2.3. In Vitro Tests of the Antioxidant Activity of the EOs Using a DPPH^•^ Assay

The scavenging DPPH^•^ free radical potential was determined in vitro according to the protocol of Nikhat et al. [14]. The experiment was carried out using a visible UV spectrophotometer at a wavelength of 515 nm. The solution of DPPH^•^ (1,1-diphenyl-di-picrylhydrazyl) at 5 M was prepared by dissolving 2.4 mg of the DPPH^•^ powder in 100 mL of ethanol. Then, 2.8 mL of the obtained solution of DPPH^•^ was mixed with 60 μL/mL of each EO or standard antioxidant (ascorbic acid) at different concentrations from 0.04 to 1.6 mg/mL. After 30 min of incubation in the dark at room temperature, the absorbance was read at 515 nm against a blank, which contained only ethanol. The positive control contained the DPPH^•^ without the extract and the obtained values were subsequently converted into inhibition percentages. The radical scavenging activity was quantified using the following equation:

AA (Scavenging effect) % = ((A_control_ − A_sample_)/A_control_) × 100

AA%: percentage of antioxidant activity;
A_control_: absorbance of the solution containing only radical DPPH^•^ solution;
A_sample_: absorbance of the sample solution to be tested in the presence of DPPH^•^.

The graph of the absorbance variation according to the concentration of extract allowed for determining the IC_50_ (concentration corresponding to the loss of 50% of free radical’s activity). 

### 2.4. Data Analysis

To conduct the statistical calculations, we used SPSS software with a level of significance of <0.05 for variance values and GraphPad Prism 5 for Windows for the means and standard deviations. Additionally, the values of IC_50_ were obtained from the third-degree polynomial trend curves in the absorbance graphs using Excel 2016.

## 3. Results and Discussion

### 3.1. Phytochemical Study

The phytochemical study consisted of the determination of the physicochemical properties of selected *Mentha* species in the form of dried matter and the EOs. These parameters allowed for evaluating their quality. However, there are few published works on the physical properties of these three mints. Moreover, the yields and the chemical composition of EOs were also determined.

#### 3.1.1. Physicochemical Properties of the Dry Matter 

The main calculated parameters of Mentha dried aerial parts are summarized in Table 1. There was a significant variation between species (*p* ≤ 0.01) and between origin regions for the same species (*p* ≤ 0.05) but the obtained values remained in compliance with the standards.

The main parameters were used to test the quality of all EOs from both regions. Most plant nutrients are optimally available to plants within this pH range (6.5 to 7.5), plus this range of pH is generally very compatible with plant root growth [15]. In addition, plants prefer mildly acidic substances. The obtained values of pH were found to range from 5.45 to 5.95. All values obtained for all species were interesting but the best ones were found for the mints from Azrou compared with those from Ifrane. Generally, they demonstrated that our samples presented good quality.

Regarding the moisture contents, all studied *Mentha* species showed significant rates, except M. suaveolens from Ifrane, whose MC reached approximately 46.68%. Moreover, the values of *M. pulegium* MC, either from Azrou or Ifrane, were found to be lower than those obtained by Tanavar et al. [16] (65.85 ± 0.43%), while those found by Tarasevičienė et al. [17] in *M. suaveolens* leaves (20.93 ± 0.12%) and stems (20.70 ± 0.34%) ranged between the higher MC of pennyroyal from Azrou and the lower MC of that from Ifrane. However, the MC of our spearmint samples from both regions seemed to be less than that of spearmint from Sudan (76.01 ± 0.03%) [18]. Moisture content is widely used in the testing of water activity of many foods. A high value implies that the plant parts may have a short shelf life since microorganisms, which cause spoilage, thrive in foods with high moisture content. The possibility of spoilage reduces with the decrease in moisture content [19]. Consequently, our species were found to be of good quality since they were dried under laboratory conditions.

Ash content is an index of mineral contents in biota. The obtained percentages were nearly similar for all studied mints, except for *M. spicata* from Ifrane (21.87%), which had the highest percentage. However, the ash content of the fresh spearmint leaves from Sudan was found to be less than ours (3.48%) [18]. As reported by Dairo et al. [20], the ash content is within the range of values (16.30–17.31%) for some vegetables [21]. Thus, in a young leaf, the ash may constitute approximately 5% of the dry weight, while in a mature leaf, it may be 15% [22]. Tarasevičienė et al. [17] concluded that the leaves of *M. suaveolens* (10.92 ± 0.018%) were richer in crude ash than the stems (8.17 ± 0.168%). Therefore, the variation in the amount of ash depends on the part of the plant, age, treatment, etc. [22].

Based on the found results, the acidity rates of three *Mentha* species from both regions ranged from 0.163% to 0.608%. The lower rates were observed for all mint species from Azrou, especially for *M. spicata* (0.163% and 0.185% for Azrou and Ifrane, respectively). However, a remarkable increase in the acidity percentage was found for fresh spearmint leaves from Sudan (2.203%) [18].

#### 3.1.2. Physicochemical Properties of the *Mentha* EOs

The extracted EOs have specific organoleptic properties. The yellow color with a lemony and very strong aromatic odor characterized the *M. pulegium* EO. The *M. suaveolens* EO was pale yellow with a fragrant odor, while the *M. spicata* EO was observed to be light yellow with a strong pleasant and fresh perfume. Similarly, the color of *M. suaveolens* from Saudi Arabia was pale yellow with a pleasant and distinct odor [23]. Moreover, the color of the tested spearmint essential oils from Pakistan was noted to be varied between yellow-green and brownish-yellow depending on their growing habitat, while the odor was similar to mint [24]. The physicochemical properties in the present analysis of *Mentha* oils served to prove their quality and their ability to be used safely. 

The Brix degree, acidity index, iodine index and peroxide value were measured and their values are summarized in Table 2. They complied with the standards, even though there were slight significant differences according to the type of species and region (*p* ≤ 0.01).

The values of the Brix index determined for all mints ranged between the lowest index 71.6 °B recorded in the *M. spicata* EO from Azrou and the highest one was observed in the *M. suaveolens* EO (81.5 °B) from Azrou. These indices were greater than those recorded for peppermint oil (24.57–27.32 °B) [25] and comparable to those measured by Radi et al. [26] for some thyme species, such as 76.62 ± 0.05 °B (*T. willdenowii*) and 85.44 ± 0.05 °B (*T. zygis*). The acid values (AVs) of the tested oils were found to be 21.91, 22.03 and 26.1 mg KOH/g for *M. pulegium*, *M. suaveolens* and *M. spicata* from Azrou, respectively, and 27.1–28.05 mg KOH/g for the mints from Ifrane. It was noted that the AVs found for *M. pulegium* and *M. suaveolens* from Azrou seemed to be similar. These results were almost comparable with the results of Salim et al. [27] and 19.7–25.2 (mg KOH/g), while those reported by Sulieman et al. [18] showed a lower value for the spearmint oil (0.0610 mg KOH/g oil).

The iodine values (the weight of iodine absorbed by 100 parts by weight of fat, where the higher the iodine value, the greater ability of oil or fat to become rancid) reached for three mint oils ranged from 103.759 to 110.160 and 106.596 to 111.122 g I/100 g from the Azrou and Ifrane regions, respectively. However, Sulieman et al. [18] found a value (0.5467 mg I/g oil) that was lower than that calculated for our spearmint oil. There is no official maximum to be expected for the PV of EOs (PV of vegetable oils <10–15 m Eq O_2_/kg) [28]. Furthermore, the PV of a fragile EO stored for a long time in a small bottle that is regularly opened with little or no protection from heat and light can easily be of the order of 2–300 m Eq O_2_/kg according to ISO 18,321 [16]. Therefore, our samples presented good PVs that oscillated between 12 and 21.6 m Eq O_2_/kg as the lower and higher amounts for the three mint species from both regions. 

Furthermore, all obtained physicochemical parameters varied between species and among the same species from the same region; this can be explained by the fact that these properties are strongly and directly affected by the distillation techniques, climatic conditions, plant varieties, regions, harvest periods, genotype, type of material and chemical composition [29]. 

#### 3.1.3. The Yield of the Mint EOs

The yields obtained from the three mints EOs varied with the region of origin. The total yield percentages of the *M. pulegium* (L.), *M. suaveolens* (Ehrh.) and *M. spicata* (L.) EOs from Azrou reached 5.9 ± 0.014%, 1.8 ± 0.009% and 2.4 ± 0.012%, respectively, whereas those from Ifrane recorded lower percentages: 2.26 ± 0.013%, 1.25 ± 0.007% and 1.18 ± 0.014%, respectively. These percentages were found to be higher than those already studied from other regions of the Middle Atlas: 5.29% for *M. pulegium* EO [30], 1.55% for *M. suaveolens* EO from M’rirt [31] and 1.54% for *M. spicata* EO from Meknes [32]. Such variation in the EO yields may be linked to the morphological and biochemical diversity of the plants attributed to different typographical conditions and environmental factors [24].

#### 3.1.4. Chemical Compositions of the *Mentha* Species EOs

**Figure 2:**
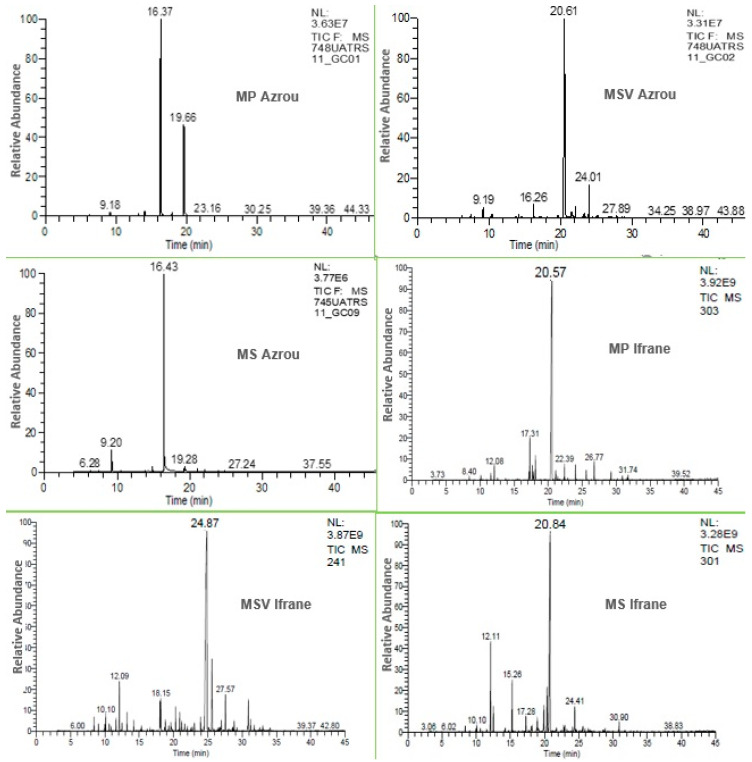
Chemical profiles of the studied mint EOs. MP Azrou: *M. pulegium*; MP Ifrane: M. *pulegium*; MSV Azrou: *M. suaveolens*; MSV Ifrane: *M. suaveolens*; MV Azrou: *M. Spicata*; MV Ifrane: *M. spicata*.

According to the chromatographic analyses of the EOs extracted from all studied mints, the chromatographic profiles were measured to allow for identifying different chemical compositions of the analyzed EOs. Some variations were observed between the three mint species and between the same species from different regions (Figure 2). 

##### Chemical Composition of the *M. Pulegium* EOs

The chromatographic analysis of *M. pulegium* essential oils allowed for identifying 26 compounds that represented approximately 99.10% of the total composition of the EO from Azrou against 33 compounds (100%) from Ifrane. Monoterpenes dominated the totality of compounds compared with sesquiterpenes in both EOs. Moreover, the EO from Azrou was richer in monoterpenes and lower in sesquiterpenes than that from Ifrane (Table 3).

The pennyroyal EO from Ifrane was dominated (70.92%) by pulegone as the main chemotype, whereas the same EO from Azrou was greatly dominated by pulegone (68.86%) and piperitenone (24.81%), accompanied by other constituents with smaller percentages, such as chrysanthenol (1.03%), thymol (1.01%), limonene (0.9%) and menth-2-en-1-ol (0.57%). However, the constituents were specific to the EO from Ifrane with significant amounts: menthone (5.03%), Cyclocitral<β-> (3.49%) and Humulene<α-> (2.17%). Moreover, some compounds were common in both EOs from Ifrane and Azrou, though presenting varying proportions, such as limonene (1.64–0.09%), caryophyllene<E> (1.19–0.04%) and caryphyllene oxide (0.47–0.09%).

The chemical composition of the pennyroyal EO was described in several works. It is characterized by the preponderance of pulegone (70–90%) accompanied by other monoterpenic ketones, such as menthone, isomenthone, piperitenone and piperitone [33]. Indeed, the described compositions of the pennyroyal EO in different countries showed the predominance of pulegone. Moroccan pennyroyal is also characterized by the dominance of this constituent with high proportions: 85.4% [34], 84.46% [35], 80.28% [36], 78.07% [37], 77.16% [38], 75.48% [39], 69.8% [40], 73.33% [41], 66.50% [42] and 65% [43]. In Egypt, pennyroyal oil contains pulegone [44]; in India, it contains 65.9–83.1% [45]; in Uruguay, it contains 73.4% [46]; and in Algeria, it contains 43.3–87.3% [47]. Others are dominated by 83.7–97.2% piperitone in Greece [48] or 70% piperitone in Austria [49]. The combination piperitone (38% and 35.56%)/piperitenone (33% and 21.18%) was found by Mahboubi et al. [50] and Derwich et al. [51]. 

Other chemical compositions resulted from the association of pulegone with piperitenone: 81.04/13.60% in Algeria [52], 71.97/26.07% and 68.86/24.81% in Morocco [35], 35.1/27.4% in Portugal [53], 27.2–49.7/19.4–57.7% in Bulgaria [54] and 32.8–75.8/5.1–35.0% in Greece [55]; for pulegone with isomenthone: 20.0–34.6/18.9–42.1% in Switzerland [56], 32.8–75.8/4.3–28.6% in Greece [48], 32.1–43.8%/41.7–52.0% in Turkey [57], 73.4–12.9% in Tunisia [58], and 61.11–17.02% in Uruguay [46]; for pulegone with menthone: 37.8–20.3% in Iran [59], 50.5/26.4% in Japan [60]; and for pulegone with isomenthol: 74.8/10.0% in Algeria [61]. On the other hand, the new association of pulegone (50.5–90%) with both menthone (1–16.9%) and isomenthone (0.6–20.7%) was described first by Sutour et al. [62]. Thereafter, EO from Morocco was characterized using the same association with proportions of 45.48, 14.2 and 7.18%, respectively [63]. However, EO from Iran was dominated by limonene (28.44%), D-carvone (18.76%) and eucalyptol (8.86%), while pulegone did not exceed 8.65% [16].

##### Chemical Composition of the *M. Suaveolens* EOs

The chromatographic analysis of EOs identified 47 compounds constituting 99.61% of the total chemical composition of the M. suaveolens EO from Azrou, whereas the same EO from Ifrane contained 35 compounds representing 100% (Table 4). Both EO of *M. suaveolens* are rich in oxygenated monoterpenes but the highest content was observed for Azrou (82.83%) against 82.69% from Ifrane, followed by the hydrocarbon sesquiterpenes with a higher percentage of approximately 10.86% from Azrou than 7.15% from Ifrane. 

Piperitenone oxide was the major constituent of the Azrou EO (74.69%), followed by low percentages of muurolene (5.53%), pulegone (2.34%), limonene (1.85%), <4a,7-β-α,α-7a>nepetalactone (1.81%), β-caryophyllene (1.68%) and piperitenone (1.17%). Similarly, the EO from Ifrane was dominated by piperitenone oxide but with a lower level (60.4%) than that from Azrou, while the constituents: <4a,7-β-α,α-7a>nepetalactone (6.09%), limonene (3.49%), Borneol (2.39%), Terpinen-4-ol (2.22%), piperitenone (1.19%), γ-terpinene (1.18%) and pinene-β (1.09%) presented higher levels than those in the EO from Azrou. Other components were also identified in the EO from Ifrane with relatively low amounts: β-caryophyllene (0.91%), limonene (0.56%), terpinene-4-ol (0.52%) and pulegone (0.47%). Differences between the oils were observed. There were compounds that were specific to the Azrou EO, such as trans-calamenene (0.77%), khusimene (0.68%) and longifolene (0.27%). On the other hand, Chamazulene (2.78%), Germacrene-D (2.74%), piperitone epoxide<trans-> (1.34%), camphene (0.48%) and sabinene (0.42%) were specific to the Ifrane species.

The chemical composition of the *M. suaveolens* oils was very variable. Numerous compounds, as well as their associations, were at the origin of a great diversity of chemical compositions described in the literature. Previous works on the chemical composition of the *M. suaveolens* (Ehrh.) EO showed that in the majority of cases, the constituents belonged to the oxygenated monoterpenes class, such as pulegone 85.47% in Beni-Mellal [64] and 50% [65]; carvone 45–64.31% in Saudi Arabia [23], 62.3% in Finland [66], 43% in Argentina [67]; cis-dihydrocarvone 43.2% [68]; menthol 48.32% in Argentina [69] and 40.50% in Morocco (Boulemane) [51]; piperitol 57.6% in Spain [70]; neo-isopulegol (52.3%) in Japan; piperitone oxide (87.3%) in Japan [69]; piperitenone oxide in Morocco: 81.69% in M’rirt [31], 71.50% in Khenifra [71], 58.26% in the northwest [72], 56% in the north [65], 53.12–54.51% in Lokkous and Azrou [72], 41.84% in the north [73] and 34% in Meknes [43]; piperitenone oxide 80.8% and 62.4% in Uruguay and Greece, respectively [46,74]; piperitone 33.03% in Oulmes, Morocco [41], and 54.91% in Algeria [75]; and linalool 35.32% in Egypt [76].

Other compositions resulted mainly from the combination of similar or variable levels of oxygenated monoterpenes. We observed the presence of piperitenone oxide (25%) and piperitone oxide cis (26%) [65], piperitenone oxide (34.4/29.36–27.79%) and piperitone oxide (40.2/19.72–31.4%) [75,77], piperitenone oxide (81.69%) and piperitenone (10.14%) [31], piperitone oxide (56.0%) and p-cymen-8-ol (20.6%) [65], piperitone oxide (41.84%) and isopulegol (11.95%), and piperitone (33.03%) and pulegone (17.61%) [41]. Some compositions were totally different from others in terms of the presence of uncommon compounds, such as 1,2-epoxyeomenthyl, 1,2-epoxyisomenthyl and 1,2-epoxyeoisomenthyl acetates (neo 66.4/26.4/22.5%, iso 10.2%, neo-iso 27.8%/10.1%) in Japan [68] or 2,4(8),6-p-menthatrien-2,3-diol (14.5%) in Cuba accompanied by Germacrene D with a proportion of 12.4% [78].

##### Chemical Composition of the *M. Spicata* EOs

Thirty-five compounds were identified in the EO from Ifrane, which represented 100% of the total chemical composition, while 96.07% of the composition of Azrou EO comprised 32 compounds. Monoterpenes were also more common in both essences than sesquiterpenes, but the EO from Azrou (88.75%) was richer in monoterpenes than that of the Ifrane EO (84.55%), which presented a higher rate of sesquiterpenes (5.27%) compared with the essence from Azrou (4.81%) (Table 5). Both oils possessed carvone and limonene as the main components. Their rates in spearmint EO from Azrou (71.56–10.50%) were much more significant than those from Ifrane (54.79–9.69%). The constituent Trans-4-caranone (2.74%) was present only in the Azrou EO plus iso-dihydrocarveol acetate (2.07%) with a moderate percentage higher than that in the EO from Ifrane. However, the latter was found to have specific constituents that reached important proportions, such as linalool acetate (7.47%), linalool (5.59%), cineol 1,8 (2.45%) and caryophyllene oxide (1.04%).

Generally, the spearmint EO was rich mainly in carvone (40 to 80%) and dihydrocuminyl acetate (10 to 12%), which are the most two major constituents responsible for the smell of the plant, along with limonene (5 to 15%); they were accompanied by dihydrocarvone, dihydrocarveol, carvylacetate and caryophyllene<α>. In other cases, carvone was accompanied by 1,8-cineole (up to 20%), pulegone (up to 50%) or 4-terpineol (up to 18%) [79]. Chemical analysis of the spearmint EOs in Morocco revealed the presence of carvone (50 to 70%) and limonene (11 to 21%) as major constituents [80]. Likewise, spearmint from Malaysia and Spain presented the same combination as ours, with carvone (41.1–78.76%) and limonene (11.50–14.4%) [77,81]. Another chemotype that characterizes the EO of *M. spicata* (L.) is piperitenone epoxide, which reaches 80% at the expense of carvone (1–2%) [82]. Other combinations from Sais Valley Morocco had carvone (73.01%), limonene (8.54%) and 1,8-cineole (6.70%) [83]. Similarly, the same species from six regions of Egypt were represented by carvone at proportions ranging from 42.23% to 73.18%, limonene from 5% to 43.84% and 1,8-cineole ranging from 4.45% to 6.05% [84]. However, the EO from Greece was characterized by its richness in carvone (71.8%) and 1,8-cineole (9%) without limonene [74]. Recently, a similar combination was reported by Anbri et al. [85] in spearmint EO from 13 sites of Morocco, which contained carvone (0.2 to 72.3%), limonene (3.1 to 18.1%) and cineole 1,8 (0.8 to 21.1%); other mentioned combinations also showed the abundance of 3-Cyclopenten-1-one,2-hydroxy-3-(3-methyl-2-butenyl)- (1 to 22.1%) in the composition.

**Figure 3:**
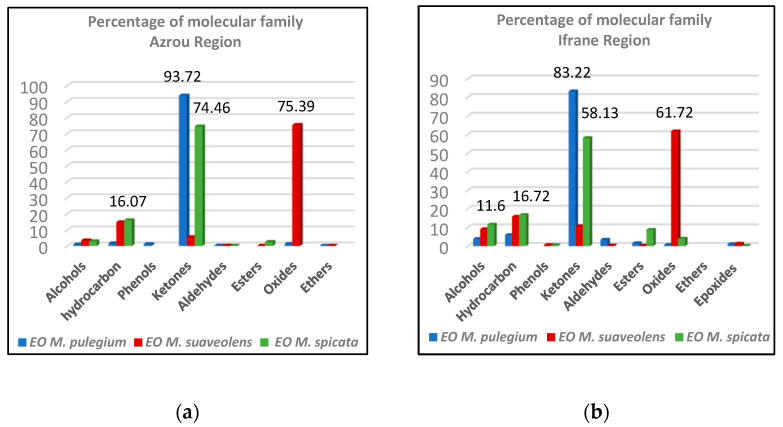
Percentages of chemical families of the studied *Mentha* species: (**a**) region of Azrou; (**b**) region of Ifrane.

The chemical compositions of EOs of the studied *Mentha* species were spread out across molecular families (Figure 3). Significant differences (*p* < 0.05) were found between the studied mints and between regions of origin for the same species in terms of the chemical families’ proportions. 

For the *M. pulegium* EOs, both were dominated by ketones, but the EO from Azrou recorded a higher percentage 93.72% than that from Ifrane (83.22%). The hydrocarbons came in second place, reaching 6% in the EO from Ifrane, while it did not exceed 1.65% in the EO from Azrou. Other chemical families were present in lower proportions, such as oxides (1.23%) and phenols (1.17%) without esters in the EO from Azrou. However, the Ifrane EO was marked by the presence of alcohols (3.9%), aldehydes (3.49%) and epoxides (1.06%), and the absence of phenols and ethers. A similarity was observed in the dominance of the oxides, hydrocarbons and ketones for *M. suaveolens* EOs with higher percentages of the oxides (75.39%) in Azrou than that of Ifrane (61.72%), while the EO from Ifrane was the richest in ketones (10.71%) and hydrocarbon (15.84%). However, the phenols and epoxides were absent in the EO from Azrou. Different from the M. suaveolens EOs, the *M. spicata* and *M. pulegium* EOs were characterized by the dominance of ketones. However, the oxides were absent in Azrou, whereas the esters, phenols and epoxides were present exclusively in the EO from Ifrane. 

Regarding the collected data about phytochemical study, we deduced that the yields and chemical compositions of the essential oils varied according to the region of origin and within the same species. This variation was due to several factors, such as the method used; the used plant parts; the products and reagents used in the extraction; the environment; the plant genotype; geographical origin; the harvest period of the plant; the degree of drying; the drying conditions; temperature and drying time; and the presence of parasites, viruses and weeds [86].

### 3.2. Antioxidant Activity of the Mint EOs

**Figure 4:**
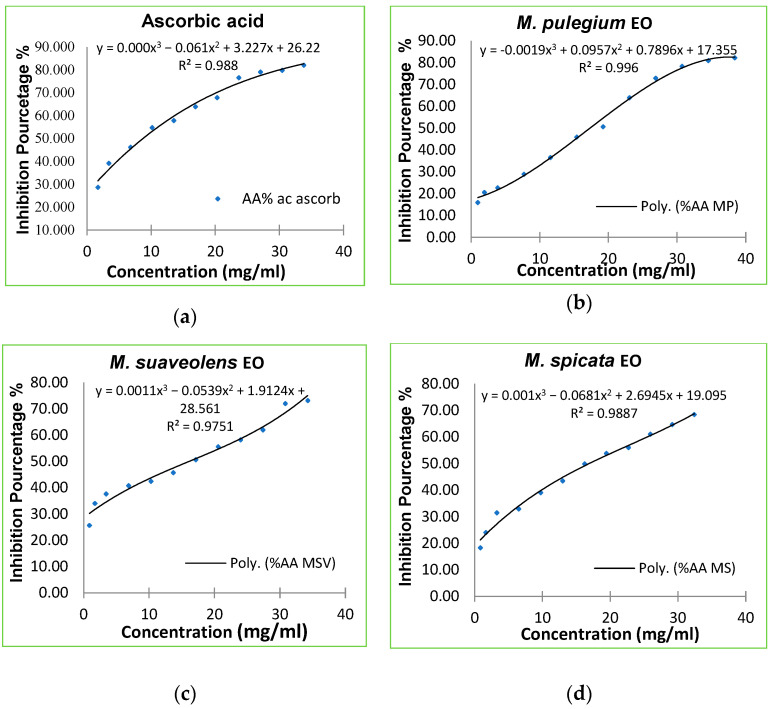
(**a**) Inhibition percentage of DPPH^•^ according to ascorbic acid concentrations; (**b**) inhibition percentage of DPPH^•^ of *M. pulegium* EO; (**c**) inhibition percentage of DPPH^•^ of *M. suaveolens* EO; (**d**) inhibition percentage of DPPH^•^ of *M. spicata* EO.

The activity of the various mint EOs toward the radical DPPH^•^ was assessed. The three mints were able to reduce the stable DPPH^•^ to the yellow-colored diphenylpicrylhydrazine. This reduction capacity was determined via a decrease in the absorbance induced by antiradical substances that occurred in the EOs. As shown below, the percentage of DPPH^•^ inhibition increased, as well as the concentration of ascorbic acid and the EOs (Figure 4). Moreover, the DPPH^•^ inhibition percentages of the EOs were found to be important but less than ascorbic acid for all the applied concentrations. They reached 82%, 73% and 70% for *M. pulegium* (L.), *M. suaveolens* (Ehrh.) and *M. spicata* (L.), respectively.

**Figure 5:**
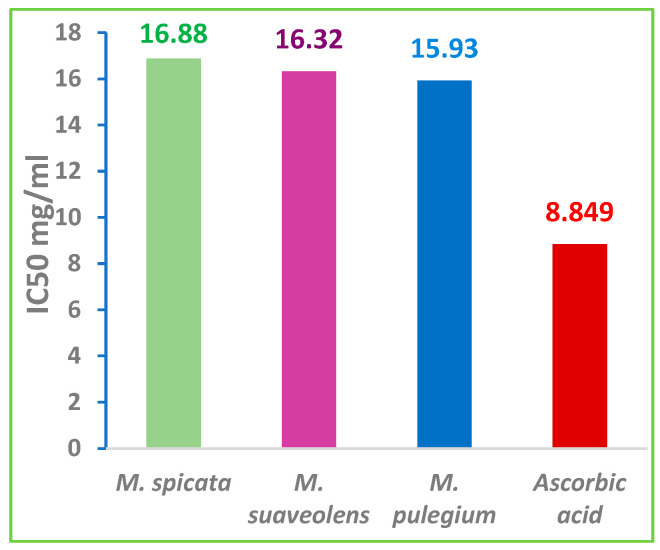
IC_50_ values of the mint EOs and ascorbic acid.

Based on the obtained IC_50_ values, the three mint EOs exhibited a significant antiradical activity compared with the ascorbic acid (IC_50_ = 8.849 mg/mL). The degree of this activity decreased as follows: ascorbic acid > *M. pulegium* > *M. suaveolens* > *M. spicata* (Figure 5). Therefore, the strongest effect was found for *M. pulegium* EO (IC_50_ = 15.93 mg/mL) and the weakest was found for *M. spicata* EO with IC_50_ = 16.88 mg/mL.

There are many reports in the literature concerning the antiradical power of mint EOs. They revealed a variation in the reaction of these EOs toward DPPH^•^. Regarding *M. pulegium* (L.), EO from Algeria showed a higher DPPH^•^ radical scavenging activity of 90.54 ± 1.5% at a concentration of 1000 µg/mL [87]. Similarly, for EOs from Tunisia and Greece, their antiradical capacity was also demonstrated, with IC_50_ values reaching 10 and 13.5 ± 0.5 μg/mL, respectively [58,88]. However, EOs extracted from Indian and Italian pennyroyal exhibited low antioxidant activities, with EC_50_ values of approximately 147.36 mg/mL and 6.2 ± 0.2 mg/mL (BHT = 0.0049 mg/mL), respectively [89,90]. This low power can be explained by a lack of antioxidants in this oil. For Mata et al. [52], this EO showed no potential for trapping the DPPH^•^, which was probably due to its weak solubility under test conditions.

The second-best inhibition of DPPH^•^ was recorded by *M. suaveolens* EO (IC_50_ = 16.32 mg/mL). Few works dealt with the antioxidant potential of this EO. The *M. Suaveolens* EO from the northwest of Morocco showed remarkable activity with IC_50_ = 64.76 ± 2.24 μg/mL against IC_50_ = 22.61 ± 1.08 μg/mL for ascorbic acid and 34.12 ± 2.13 μg/mL for Trolox [74]. EO from Egypt exerted a moderate in vitro antiradical potential, with IC_50_ of 200 μg/mL compared with ascorbic acid (7.5 μg/mL) [53]. Thereafter, two studies confirmed this moderate activity [91,92].

Spearmint oil also presented an antiradical potency but this was lower than the *M. pulegium* and *M. suaveolens* EOs. Our results concorded with previous studies. The inhibition capacity of Tunisian spearmint oil was found to be higher (IC_50_ = 3 μg/mL) than the positive control BHT (IC_50_ = 11.5 μg/mL) [93]. This oil seemed to be a more powerful antioxidant than that reported by Dhifi et al. [94], which had an IC_50_ equal to 10 μg/mL. As for Mkaddem et al. [95], they deduced that the investigated spearmint oil had a moderate reducing power (IC_50_ = 3476.3 mg/L) compared with ascorbic acid (4.4 ± 0.2 mg/L). Similarly, spearmint EO from Portugal showed antiradical activity that reached 31.45% [96], while that from the same origin revealed no antioxidant activity [95]. Moreover, a spearmint EO from Algeria had a significant scavenging activity expressed by an IC_50_ close to 208.495 ± 4.247 μg/mL [97]. Recently, Alsaraf et al. [98] found the promising antioxidant activity of a spearmint EO from Oman with IC_50_ = 26.64 μg/mL.

We note that our investigated oils presented the important ability to eliminate the radical DPPH^•^. This interesting property might be related to the chemical composition of EOs and mainly to the phenolic compounds that may play an important role in inhibiting the auto-oxidation of oils [6]. Consequently, the tested mint EOs were rich in oxygenated monoterpenes and sesquiterpenes, such as pulegone (68.86%) and thymol (1.01%) for the *M. pulegium* EO, carvone (71.56%) and limonene (10.50%) for the spearmint EO, and, finally, piperitenone oxide for the *M. suaveolens* EO. Moreover, other compounds found in lower amounts also showed their contribution to the antioxidant activity: such as β-pinene, ɤ-terpinene, α-terpineol and spathulenol [93,96,99]. On the other hand, the higher antioxidant effect of the *M. pulegium* EO compared with the other tested EOs, as indicated in many mint species [89,100], may be explained by the high content of pulegone and by the presence of phenolic compounds, such as thymol, which acted as potential antioxidant [4,99,101].

## 4. Conclusions

The physical properties and chemical compositions of the three studied mints showed considerable variations according to the harvesting region. The data obtained revealed that the region was a crucial factor in the bioactive composition. The *M. pulegium* EO from Azrou was dominated by pulegone (68.86%) and piperitenone (24.81%) but the same EO from Ifrane contained pulegone as the main component (70.92%). Piperitenone oxide (74.69–60.3%), carvone (71.56–54.79%) and limonene (10.5–9.69%) were the major constituents with different rates for *M. suaveolens* and *M. spicata* from Azrou and Ifrane, respectively. Moreover, the identification of the physicochemical parameters of these oils led to demonstrating the quality that could be useful in future studies for medicinal or pharmaceutical purposes. The results also indicated that the mint EOs exhibited remarkable antioxidant properties that were comparable to ascorbic acid, especially the EO of *M. pulegium*, which was the most active. This activity was attributed to the occurrence of phenolic content in these EOs. These promising results showed that they have the potential to be a natural alternative to synthetic antioxidants in order to enhance the shelf life and safety of food.

## Figures and Tables

**Table 1:** Quality control parameters of dry matter.

Species	pH		MC (%)		Acidity (%)		Ash Content (%)	
	Azrou	Ifrane	Azrou	Ifrane	Azrou	Ifrane	Azrou	Ifrane
*M. pulegium*	5.45 ± 0.029	5.78 ± 0.027	16 ± 0.017	13.94 ± 0.020	0.210 ± 0.002	0.294 ± 0.003	11.25 ± 0.011	11.86 ± 0.013
*M. suaveolens*	5.67 ± 0.011	5.95 ± 0.016	17 ± 0.013	23.44 ± 0.016	0.590 ± 0.003	0.608 ± 0.004	11.43 ± 0.013	11.72 ± 0.022
*M. spicata*	5.58 ± 0.013	5.82 ± 0.022	20 ± 0.009	46.67 ± 0.013	0.163 ± 0.001	0.185 ± 0.002	18.89 ± 0.016	21.87 ± 0.018

**Table 2:** Parameters of quality control of mints EOs.

Parameters	*M. pulegium EO*		*M. suaveolens EO*		*M. spicata EO*	
	Azrou	Ifrane	Azrou	Ifrane	Azrou	Ifrane
Brix index (°B)	72.8 ± 0.099	79.23 ± 0.111	80.7 ± 0.212	81.43 ± 0.222	71.6 ± 0.130	72.30 ± 0.133
Acidity index (mg KOH/g)	21.91 ± 0.008	28.07 ± 0.011	22.03 ± 0.019	28.06 ± 0.029	26.1 ± 0.036	27.10 ± 0.038
Iodine index (g I/100 g)	110.160 ± 0.001	110.17 ± 0.001	107.354 ± 0.018	106.61 ± 0.019	103.759 ± 0.001	111.12 ± 0.002
Peroxide value (m Eq O_2_/kg)	12.01 ± 0.136	14.03 ± 0.178	20.9 ± 0.129	21.60 ± 0.133	18.4 ± 0.172	19.27 ± 0.178

**Table 3:** Chemical composition of *M. pulegium* (L.) EOs from the Middle Atlas.

Compound	Calculated RI	MP Area (%)	
		Azrou	Ifrane
α-pinene	939	0.17	0.35
β-pinene	979	0.15	-
Myrcene	987	-	0.48
Meta-mentha-1(7), 8-diene	1000	0.02	-
O-cymene	1026	0.07	-
Octanol<3->	1027	-	0.77
Limonene	1029	0.90	1.64
Cineole<1,8->	1031	-	0.23
Para-mentha-3,8-diene	1072	0.01	0.17
Thujone<Trans->	1136	-	0.13
Menth-2-en-1-ol<Trans-ρ->	1140	0.57	-
Octanolacetate<3->	1143	-	0.08
Benzylacetate	1162	0.07	-
Chrysanthenol<cis->	1164	1.03	-
Menthone<iso->	1170	-	1.66
Menth-2-en-1-ol <cis-ρ->	1184	-	1.09
Terpineol< α>	1188	0.17	-
Menthone	1198	-	5.03
Levomenthol	1203	-	1.37
Cyclocitral<β->	1212	-	3.49
Pulegol<Trans>	1214	0.19	-
Coahuilensolmethylether	1221	0.16	-
Verbenone	1249	-	0.1
Pulegone	1237	68.86	70.92
Piperitone	1252	0.07	-
Piperitoneepoxide<cis->	1255	-	0.1
Perilla-aldehyde	1271	0.21	-
Thymol	1290	1.01	-
Carvacrol	1299	0.04	--
Menthylacetate	1300	-	1.55
Piperitoneepoxide<trans-> -	1308	-	0.42
p-vinyl-guaiacol	1309	0.13	-
Menth-1-en-9-ol <ρ->	1316	-	0.08
Tetrahydrojasmone<trans->	1339	-	1.89
Piperitenone	1343	24.81	2.03
Piperitoneepoxide<trans->	1355	-	0.26
Caryophyllene<Z->	1408	0.11	-
Caryophyllene<E>	1419	0.04	1.19
α–Guaiene	1439	0.08	-
Ionol<α-(E)->	1380	-	0.59
Ionone <dihydro-α->	1435	-	0.10
Humulene<α->	1454	-	2.17
Ionone<Trans-β>	1486	-	0.13
Dihydroagarofurane<4-Epi-Cis >	1499	0.04	-
Germacrene D-4-ol	1575	0.09	-
Nepetalactone<4aα,7α,7aβ->	1566	-	1.21
Caryophyllene oxyde	1583	0.09	0.47
Humulene epoxide II	1606	-	0.28
Himachalol	1653	0.01	-
Oxygenated monoterpenes		96.89	85.39
Hydrocarbon monoterpenes		1.34	3.41
Oxygenated sesquiterpenes		0.19	2.8
Hydrocarbon sesquiterpenes		0.31	3.36
Others		0.37	5.12
Total (%)		99.10	100

**Table 4:** Chemical composition of *M. Suaveolens* (L.) EOs from the Middle Atlas.

Compound	Calculated RI	Area (%)	
		Azrou	Ifrane
α-pinene	939	0.36	0.88
Camphene	954	-	0.48
Sabinene	983	-	0.42
β-pinene	979	0.65	1.09
Meta-mentha-1(7),8-diene	1000	0.18	-
Myrcene	1001	-	0.44
Octanol<3->	1008	-	0.3
α-Terpinene	1017	0.07	0.71
p-cimene	1024	0.13	
Limonene	1029	1.85	3.49
Cineole<1,8->	1052	-	0.55
γ -terpinene	1059	0.13	1.18
Cis-sabinene hydrate	1070	0.53	0.67
Trans-sabinene hydrate	1098	0.06	-
1-octen-3-yl-acetate	1112	0.13	0.32
Dehydro-sabinacetone	1120	0.05	-
4-acetyl-1-methyl cyclohexene	1137	0.08	-
Nopinone	1140	0.05	-
Sabina Ketone	1158	-	0.31
Borneol	1169	0.27	2.39
Terpinen-4-ol	1177	0.71	2.22
p-cymen-8-ol	1182	0.12	
α-Terpineol	1188	0.25	0.77
MethyletherCoahuilensol	1221	0.14	
Pulegone	1237	2.34	1.7
Piperitoneepoxide<trans->	1221	-	1.34
2-Allyl-p-cresol	1255	-	0.76
CarvoneOxide Cis	1263	0.44	0.87
Carvone	1242	-	0.61
Geranial	1267	-	-
Perillaaldehyde	1271	0.17	-
Carvacrol	1298	-	0.63
Cinnamaldehyde<α-methyl>	1317	-	0.33
Piperitenone	1343	1.17	1.19
PiperitenoneOxide	1368	74.69	60.3
Daucene	1381	0.11	-
Bourbonene<β->	1387	-	0.25
β-Elemene	1390	0.16	-
<4a-α,7-β,7a-α>nepetalactone	1391	1.81	6.9
Calamenene<cis->	1400	-	0.68
Longifolene	1407	0.27	-
β-Caryophyllene	1419	1.68	-
Cis-muurola-3,5-diene	1450	0.09	-
Spirolepechinene	1451	0.16	-
Khusimene	1455	0.68	-
cis-cadina-1(6),4-diene	1463	0.81	-
Muurola-4(14),5-diene <cis->	1464	-	0.7
Germacrene D	1478	-	2.74
γ -Muurolene	1479	5.53	-
γ -Amorphene	1495	0.30	-
Aciphyllene	1501	0.10	-
γ -cadinene	1513	0.11	-
Trans-calamenene	1522	0.77	-
α-cadinene	1538	0.09	-
Spathulenol	1578	0.60	0.31
Oxyde de Caryophellene	1582	0.26	-
Globulol	1590	0.23	
Ledol	1602	-	-
Viridiflorol	1603	-	0.86
Cubenol<1-epi->	1618	-	0.46
1,10-di-epi-Cubenol	1619	0.43	-
Chamazulene	1625	-	2.78
10-epi-α-cadinol	1640	0.28	-
Torreyol	1646	0.05	-
*α*-cadinol	1654	0.35	0.37
Germacra-4 (15), 5,10(14) trien-1-α-ol	1686	0.07	-
Shyobunol	1689	0.10	-
Oxygenated monoterpenes		82.83	82.69
Hydrocarbon monoterpenes		3.37	7.51
Oxygenated sesquiterpenes		2.37	2
Hydrocarbon sesquiterpenes		10.86	7.15
Others		0.18	0.65
Total (%)		99.61	100

**Table 5:** Chemical composition of *M. spicata* (L.) EOs from the Middle Atlas.

Compound	Calculated RI	Area (%)	
		Azrou	Ifrane
*α*-pinene	933	0.37	0.56
Sabinene	983	-	0.27
*β*-pinene	975	0.58	0.65
Myrcene	1001	-	0.26
Octanol<3->	1027	-	0.24
Cineole 1,8	1028	-	2.45
Limonene	1029	10.50	9.69
*p*-mentha-3,8-diene	1065	0.79	2.45
Sabinene hydrate <cis->	1070	-	0.36
Linalooloxide<cis->	1095	-	0.15
Terpinolene	1097	0.10	-
Linalool	1098	-	5.59
Menthone	1152	-	1.49
Menthone<iso->	1153	-	0.19
Borneol	1165	0.78	0.70
Terpinen-4-ol	1177	0.65	0.56
*α*-terpineol	1191	0.12	-
Trans-4-caranone	1195	2.74	-
Trans-carveol	1227	0.22	3.54
Dihydrocarvone cis	1202	-	1.66
Dihydrocarveolneo	1203	-	0.43
Pulegone	1238	0.16	-
Carvone	1242	71.56	54.79
Linaloolacetate	1251	-	7.47
*p*-cymen-7-ol	1287	0.08	-
Carvoneoxide	1262	-	0.42
Thymol	1290	-	0.14
*ɣ*-Terpinen-7-al	1292	0.10	-
Carvacrol	1299	-	0.41
Iso-dihydrocarveolacetate	1329	2.07	0.57
Trans-carvylacetate	1342	-	0.25
cis-carvylacetate	1364	0.44	0.52
*α*-Yalengene	1375	0.08	-
*β*-Bourbonene	1384	1.04	2.84
*β*-Elemene	1391	0.09	0.30
Caryophyllene (E)	1418	-	0.87
*β*-Caryophyllene	1418	0.76	-
*β*-Coparene	1428	0.16	-
6,9-Guaiadiene	1444	0.15	-
Spirolepechinene	1452	0.12	-
Cis-Cadina-1(6),4-diene	1462	0.15	-
Cis-Muurola-4(14),5-diene	1466	0.11	-
Germacrene D	1479	0.61	0.35
Muurolene<γ->	1468	-	0.45
Farnesene<(Z)-β->	1476	-	0.13
Trans-calamenene	1522	0.33	0.35
Spathulenol	1575	0.14	0.18
Trans-Sesquisabinene hydrate	1579	0.13	-
Caryophylleneoxide	1583	-	1.04
Humulene epoxide II	1588	-	0.21
Globulol	1590	0.23	-
1,10-di-epi-Cubenol	1614	0.17	-
Hinesol	1640	0.30	-
α-Cadinol	1654	0.24	-
Oxygenated monoterpenes		76.41	72.88
Hydrocarbon monoterpenes		12.34	11.67
Oxygenated sesquiterpenes		1.21	1.43
Hydrocarbon sesquiterpenes		3.6	5.29
Others		2.51	8.81
Total (%)		96.07	100

## Data Availability

The data are available from the corresponding author.
